# Mean-field model of melting in superheated crystals based on a single experimentally measurable order parameter

**DOI:** 10.1038/s41598-021-97124-7

**Published:** 2021-09-09

**Authors:** Nikita P. Kryuchkov, Nikita A. Dmitryuk, Wei Li, Pavel V. Ovcharov, Yilong Han, Andrei V. Sapelkin, Stanislav O. Yurchenko

**Affiliations:** 1 Bauman Moscow State Technical University, 2nd Baumanskaya street 5, Moscow, Russia 105005; 2 Department of Physics, Hong Kong University of Science and Technology, Clear Water Bay, Hong Kong SAR China; 3 School of Physics and Astronomy, Queen Mary University of London, London, E1 4NS England

**Keywords:** Phase transitions and critical phenomena, Colloids, Structure of solids and liquids, Nonlinear phenomena, Statistical physics

## Abstract

Melting is one of the most studied phase transitions important for atomic, molecular, colloidal, and protein systems. However, there is currently no microscopic experimentally accessible criteria that can be used to reliably track a system evolution across the transition, while providing insights into melting nucleation and melting front evolution. To address this, we developed a theoretical mean-field framework with the normalised mean-square displacement between particles in neighbouring Voronoi cells serving as the local order parameter, *measurable experimentally*. We tested the framework in a number of colloidal and *in silico* particle-resolved experiments against systems with significantly different (Brownian and Newtonian) dynamic regimes and found that it provides excellent description of system evolution across melting point. This new approach suggests a broad scope for application in diverse areas of science from materials through to biology and beyond. Consequently, the results of this work provide a new guidance for nucleation theory of melting and are of broad interest in condensed matter, chemical physics, physical chemistry, materials science, and soft matter.

## Introduction

The phenomenon of melting is ubiquitous all around us, from atomic and molecular to protein and colloidal systems, and extends well beyond materials science. Hence formulation of microscopic-scale melting criteria has received significant attention for at least last 100 years. One of the most widely used microscopic approaches is due to Frederic Lindemann^1^ and, in particular, its reinterpretation by Gilvarry^2^. What is now known as Lindemann criterion states that melting takes place when the mean-square displacements (MSD) of atoms from their position reaches a certain proportion (typically 0.1–0.15) of the interatomic distance. The popularity of the criterion is due its simplicity and intuitive appeal, but there are also significant shortcomings including poor precision in predicting melting point and not including explicitly the liquid state^3^. Consequently, a number of criteria that can be traced back to Gilvarry’s work have been introduced^4^, particularly with the development of modern experimental and computational methods that provide access to the atomic displacements. Furthermore, the original Lindemann approach is unsuitable for 2D systems where MSDs of particles are diverging logarithmically. To resolve this problem, modified Lindemann criterion for the relative MSDs between the nearest neighbours in crystalline lattice was proposed in Ref.^5,6^.

Melting as a dynamic instability if crystalline lattice was also considered by Max Born^7^. According to the Born’s dynamic criterion, the melting point corresponds to the zero value of shear modulus. Thus, the Lindemann and Born criteria (and their various modifications) approach description of melting from two different sides – microscopic and macroscopic behaviour respectively. Typically, these two approaches are considered separately, despite well-known coupling of structure, dynamics, and thermodynamics in fluids and crystals near melting line. Furthermore, in the framework of the two approaches the details of structural changes and collective dynamics remain elusive for number of key phenomena, including details of nucleation mechanism, melting front kinetics, and system behaviour near melting point.

At the same time, particle-resolved experiments with model systems, accompanied by molecular dynamic (MD) simulations, allow to observe in unprecedented details phase transitions in different regimes, from weakly to strongly non-equilibrium ones^8^. Single particle-resolved studies with colloids have allowed to investigate a wide range of phenomena^9,10^, including melting and crystallisation^11–21^, solid-solid phase transitions^21–23^, condensation and critical phenomena^24^, gelation and glassy state^25–27^, supercooled fluids^28^. In particular, important studies were performed with thermally-sensitive N-isopropylacrylamide microgel (NIPAm) colloidal spheres (whose diameters drop with increase in temperature), including premelting at defects^11^, grain-boundary roughening^29^, polycrystalline structures^30^, two-step nucleation during solid-solid phase transitions^31^, and melting in superheated crystals^32^. Similarly to colloids, particle-resolved studies with complex plasmas (charged microparticles in ionised gas^33^) have successfully allowed to investigate melting and crystallization^34–36^, spinodal decomposition^37,38^, glassy state^39,40^, evolution of crystalline domains^41^, excitations in fluids^42,43^, thermal activation and propagation of nonequilibrium melting fronts^44–46^ (closely related to dissipative phase transitions between thermally-activated and nonactivated states^47,48^). Thus, significant insights into generic mechanisms of crystal melting can be obtained with model systems^8,33^. The particle-resolved studies with colloidal systems can also be applied to globular protein solutions^10,49^ extending the capabilities even further.

In this context, a general theoretical framework that can link model systems and real materials with particle-resolved (e.g. MD) simulations would be of significant practical interest. However, several questions that arise from the particle-resolved studies must be addressed: Could such a model be based on microscopic parameters experimentally accessible in real (i.e. atomic and molecular) systems? Could these phenomena be described in the same manner in systems with different dynamic regimes (e.g. Brownian in globular proteins and colloids and Newtonian in dusty plasma and atomic systems)? How far (if at all) the physical analogy between nonequilibrium phenomena in weakly damped (complex plasmas) and overdamped (colloids, proteins) systems extends to melting in real atomic systems?

Motivated by these open questions, we developed a new mean-field theoretical framework to describe melting on a microscopic scale and tested it by studying fronts propagating in superheated crystals during nuclei growth, as well as with numerical analysis of nucleation process. Our model is based on a local order parameter we introduced—mean-square displacement reformulated to include particles in neighboring Voronoi cells, thus introducing local correlations into the mean-field model. The collective dynamics is taken into account through kinetic constants (damping), with a stochastic source providing thermal fluctuations to obey the fluctuation-dissipation theorem. We found that our approach provides accurate description of melting of superheated colloidal (NIPAm) systems and model crystals in atomistic MD simulations, despite significant differences in their dynamics (Brownian vs Newtonian). We established that the proposed model exhibits rich behaviour including bifurcation (attributed to the homogeneous nucleation process) at the initial stages of melting and is also demonstrates analogy with the model^44,48^ describing thermal evolution in chemically-reactive media and in complex (dusty) plasma crystals, suggesting broad scope for the model applications, from atomic and molecular to colloid and globular protein systems.

## Results and discussion

### Self-consistent mean-field model of $\lambda ^2$-field evolution

**Figure 1:**
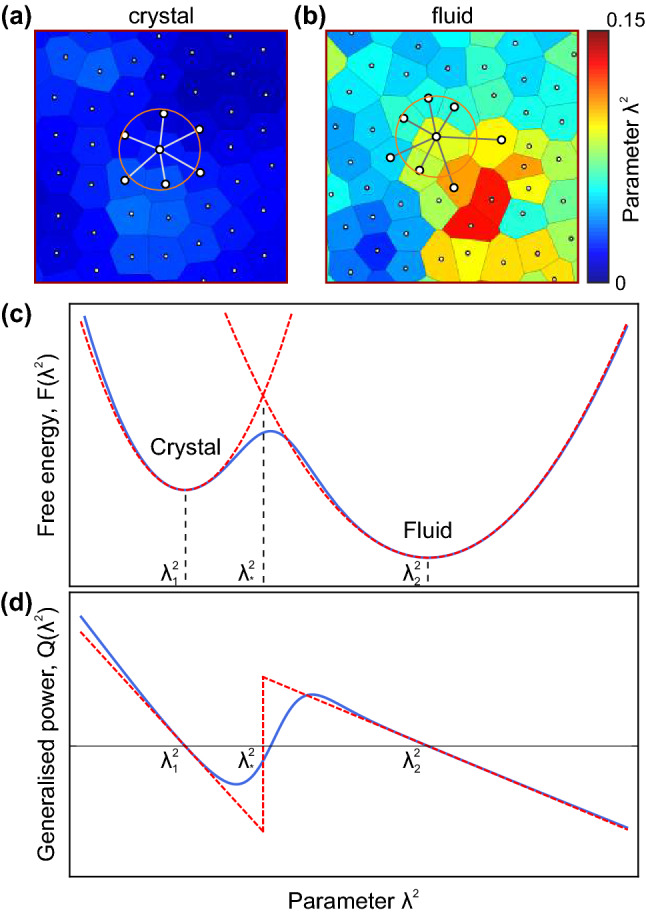
Sketches toward the proposed self-consistent $\lambda ^2$-model: (**a**) and (**b**) the examples—the crosses of the system in crystalline and fluid state (taken from our MD simulations). Voronoi cells are coloured in accordance with corresponding values of $\lambda ^2$-parameter. (**c**) and (**d**) Schematically illustrate free energy dependence $F(\lambda ^2)$ (in homogeneous system) and the generalised power $Q(\lambda ^2)$ conjugated to the $\lambda ^2$-field, shown with blue solid lines. The dashed red lines illustrate the stepwise approximations (4) and (6).

Examples of crystalline and fluid structures are illustrated in Fig. 1a, b. Here, the white points are particles, the Voronoi cells are shown with solid grey lines, the cells are coloured in accordance to $\lambda ^2$-value—the normalised mean-square displacement between particles in neighboring Voronoi cells^50^. In Ref.^50^, to characterise the local disorder and to differentiate between condensed (liquid or solid) phases, we proposed an approach based on the analysis of Voronoi cells. Within the approach, the system is split into Voronoi cells to calculate the following parameter

1 $$\begin{aligned} \sigma _{i} =\frac{1}{a_i N_{ni}}\sqrt{\sum _{j<k}^{N_{ni}}{(r_{ij}-r_{ik})^2}/2}, \quad r_{ij}=|\mathbf {r}_i-\mathbf {r}_j|, \end{aligned}$$

where $\mathbf {r}_i$ is the radius-vector of the *i*-th particle, $N_{ni}$ is the number of the neighbouring cells, $a_i = \sqrt{S_i/\pi }$ is the characteristic radius, $S_i$ is the area of Voronoi cell. Then, in order to suppress strong local thermal fluctuations, the averaging with between neighbouring Voronoi is performed as follows^50^,

2 $$\begin{aligned} \lambda _{i} = \frac{1}{N_{ni}+1}\left( \sigma _{i}+\sum _{j=1}^{N_{ni}}{\sigma _{j}}\right) . \end{aligned}$$

As a result, we obtain the standard deviation $\lambda _i^2$ of the distances between the neighbouring particles in *a physically-small volume* in the vicinity of the *i*-th particle. Crucially, this new $\lambda _i^2$ metric, while retaining the information about the local particle displacements, works equally well for characterisation of both solid and liquid phases of a system since it characterises the local disorder in a physically small local volume^50^. In crystals, $\lambda ^2$ is related to the Lindemann parameter for the neighbouring particles^5^ because $\lambda ^2 \propto \sigma _\Vert ^2$, where $\sigma _\Vert ^2$ is the longitudinal component of the mean-squared displacement of the nearest particles. Moreover, $\sigma _\Vert ^2$ plays an important role in the calculation of the first correlation peak in crystals^51–55^. After melting, the crystalline lattice is broken, but the Voronoi decomposition is still applicable in liquid despite particle diffusion. Thus, in the case of systems with repulsion, the growth in $\lambda ^2$ is provided by (i) an increase in temperature or (ii) a decrease in density. Whereas the former mechanism plays the central role in systems with soft repulsion between particles (e.g., in soft crystals at low temperatures $\lambda ^2 \propto T$), the latter one is decisive in hard-sphere-like systems (such as NIPAm colloids), whose collective dynamics is driven by the particle volume fraction. Importanly, as we show below, in both cases, $\lambda ^2$ plays the role of order parameter, and, in these terms, the melting is the transition from low-$\lambda ^2$ (crystalline) to high-$\lambda ^2$ (liquid) state.

Consider a weakly inhomogeneous spatial field $\lambda ^2$. The $\lambda ^2$-parameter is nonconcervative and, therefore, its evolution is then determined by the following time-dependent Langevin equation^56^:

3 $$\begin{aligned} \frac{\partial \lambda ^2}{\partial t} = -\Gamma \frac{\delta \mathcal {F}}{\delta \lambda ^2} + \varepsilon ^{1/2}\xi (t,\mathbf {r}), \end{aligned}$$

where $\Gamma$ is the generalised viscosity, $\mathcal {F}$ is the free energy functional of the system, $\langle \xi (t,\mathbf {r})\xi (t',\mathbf {r}')\rangle = \delta (t-t')\delta (\mathbf {r}-\mathbf {r}')$, and $\varepsilon = 2k_BT\Gamma$. The last term in Eq. (2) describes thermal fluctuations of the $\lambda ^2$-field related to the fluctuation-dissipation theorem. One should note here that Eq. (3) is related also to Ref.^57^, a seminal work, where it was shown that under certain assumptions, the microscopic master equation for cluster formation can be coarse-grained into a diffusive-type dynamics in the cluster size space.

The free energy functional is $\mathcal {F}[\lambda ^2] = \int {d\mathbf {r}\;F[\lambda ^2]}$, while in the second order approximation

4 $$\begin{aligned} F[\lambda ^2] = F_{{1,2}}^{(0)}+\frac{1}{2}A_{1,2}\left( \lambda ^2-\lambda _{1,2}^2\right) ^2 + \frac{1}{2}\alpha _{1,2}\left( \nabla \lambda ^2\right) ^2, \end{aligned}$$

where $F_{1,2}^{(0)}$ is the energy of homogeneous state (1 or 2), *A* and $\alpha$ are the positive coefficients of the expansion^56^, and the indices 1 and 2 correspond to the crystalline or fluid state, at $\lambda ^2 \lessgtr \lambda _*^2$, respectively. Here, $\lambda _*^2$ is the threshold value and we assume that $F_{{1}}^{(0)}>F_{{2}}^{(0)}$ for the case we consider.

Using Eqs. (3) and (4) we readily obtain

5 $$\begin{aligned} \frac{\partial \lambda ^2}{\partial t} = \chi _{1,2} \nabla ^2\lambda ^2 + Q(\lambda ^2) + \varepsilon ^{1/2}\xi (t,\mathbf {r}), \end{aligned}$$

where $\chi _{1,2} = \alpha _{1,2}\Gamma$ is the generalised $\lambda ^2$-diffusivity, and $Q(\lambda ^2)$ is the generalized source of $\lambda ^2$-field,

6 $$\begin{aligned} Q(\lambda ^2) = \left\{ \begin{array}{ll} -\gamma _{1}\left( \lambda ^2-\lambda _{1}^2\right) , &{} \lambda ^2 < \lambda _*^2;\\ -\gamma _{2}\left( \lambda ^2-\lambda _{2}^2\right) , &{} \lambda ^2 > \lambda _*^2,\\ \end{array} \right. \end{aligned}$$

where $\gamma _{1,2} = \Gamma A_{1,2}$. Equation (5) exhibits a remarkable analogy with temperature evolution in chemically-reactive media^58^ and coincides with that for kinetic temperature studied in Refs.^44,45,48^ during analysis of propagating of nonequilibrium melting fronts in monolayer dusty plasma crystals. Note, the proposed model can be generalised to account for the energy release at the interface during melting front propagation by adding a coupled equation for temperature evolution, similar to that reported in Ref.^59^. However, the temperature in our experiment was constant, since it is determined by the solvent temperature (Brownian thermostat). Due to this, the heat release is assumed to be negligible in hard-sphere-like systems, the temperature was approximately constant, and the processes were determined only by the configurational change in free energy (described by $\lambda ^2$-parameter). Therefore, the model (5) is sufficient for the scope of the present work. At the same time, the combination of equations for order parameter and temperature change due to the release of latent heat could be the next step, and we leave it for future studies.

The energy (4) for the homogeneous case and the corresponding generalised power $Q(\lambda ^2)$ are illustrated in Fig. 1c,d. We see in Fig. 1d that the system can exist for a long time in the vicinity of stable states with $\lambda ^2 = \lambda _{1,2}^2$, whereas the threshold value $\lambda ^2 = \lambda _*^2$ corresponds to the unstable point. Below we show that solutions of Eq. (5) explain two important phenomena studied in the present paper: (i) propagating self-similar fronts of melting in superheated crystals of particles moving in Brownian or Newtonian dynamic regimes and (ii) bifurcation behaviour of $\lambda ^2$-fluctuations (melting nuclei), and (iii) homogeneous nucleation in a superheated crystal.

Consider *propagating melting fronts* in a system, neglecting the effects of thermal noise and assuming that the curvature of the front is negligible: i.e. we assume $\epsilon \simeq 0$ and write $\nabla ^2 = \partial ^2/\partial r^2$ in Eq. (5). The self-similar profile (running wave of melting) is then described by the function $\lambda ^2(t-r/v_{\rm{fr}})\equiv \lambda ^2(\tau )$ (here, $v_{\rm{fr}}$ is the melting front velocity), which obeys the equation

7 $$\begin{aligned} \frac{\chi _{1,2}}{v_{\rm{fr}}^2} \frac{d^2 \lambda ^2}{d\tau ^2} -\frac{d \lambda ^2}{d \tau } -\gamma _{1,2}(\lambda ^2-\lambda _{1,2}^2) =0, \end{aligned}$$

considering $\lambda ^2(\tau )$ and its derivative $d\lambda ^2/d \tau$ should be continuous at the point $\tau =0$, where $\lambda ^2=\lambda _*^2$. This equation is identical to that arising in the problem of nonequilibrium melting in complex plasma crystals, hence the solution of Eq. (7) is also the same^44,48^:

8 $$\begin{aligned} \frac{\lambda ^2(\tau )-\lambda ^2_1}{\lambda ^2_*-\lambda ^2_1}= \left\{ \begin{array}{ll} e^{p_1 \tau }, &{} \tau < 0;\\ 1+\left( 1-e^{-p_2\tau }\right) p_1/p_2 , &{} \tau > 0,\\ \end{array} \right. \end{aligned}$$

where $p_{1,2} = \left( \sqrt{1+4\gamma _{1,2} \chi _{1,2}/v_{\rm{fr}}^2}\pm 1\right) v_{\rm{fr}}^2/2\chi _{1,2}$ are the rates of the exponential branches before and after the melting front. At $\tau \gg 1$, $\lambda ^2(\tau ) \rightarrow \lambda _2^2$, hence we obtain the condition $\left( \lambda _2^2-\lambda ^2_1\right) /\left( \lambda ^2_*-\lambda ^2_1\right) = (1+p_1/p_2)$. The velocity of melting front and the rates $p_{1,2}$ (unknown *a priory*) are determined in a complicated manner by $\lambda ^2$-diffusion, governed by the collective dynamics of particles in crystal and fluid, as well as by specificity of interparticle interactions and the difference of chemical potentials at the fluid-solid interface^13^. In the following sections, we test the model introduced above against bulk NIPAm colloidal crystal and atomistic MD simulations to demonstrate that it describes well evolution of the $\lambda ^2$ field and propagation of the meting fronts in superheated crystals.

### Direct observation of self-similar profile of steady melting fronts in superheated colloids

The first observation following from Eq. (5) (with $\varepsilon = 0$) is that the self-similar profile $\lambda ^2(\tau )$ is a combination of two *exponential branches* in Eq. (8). To test this, we analysed the experiment with bulk NIPAm colloids explained in "Materials and methods". This colloidal system is a good model for hard-sphere-like system^13,32^. The hard-sphere interaction represents the simplest interaction between two particles with the only restriction is that two particles can not penetrate into each other. All possible configurations have zero potential energy, implying that the free energy is entirely governed by entropy. That means, the only control parameter to govern phase state (and other properties of the system) is the particle volume fraction $\phi = N V_p/ V$, representing the dimensionless analogue of particle number density (here, *N* is the number of particles, $V_p$ is the single particle volume, and *V* is the total system volume). At the same time, the volume fraction can be tuned with laser heating of NIPA colloids in the experiment.

**Figure 2:**
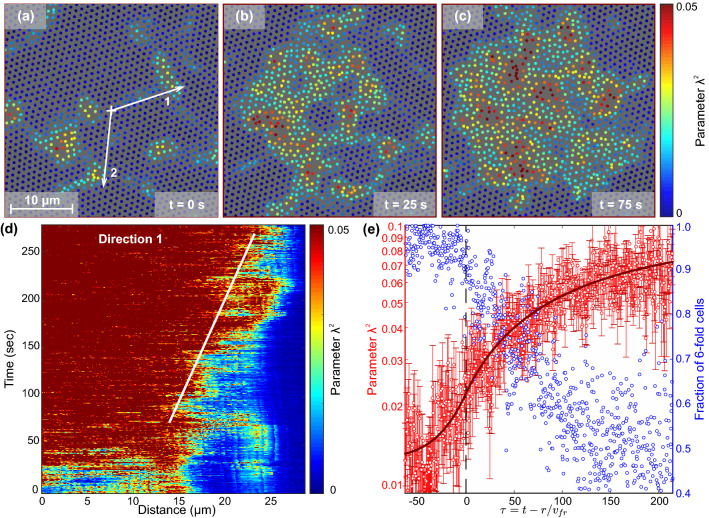
Experimental reveal of the self-similar $\lambda ^2$-profile in the propagating melting front in bulk colloidal crystal at intermediate superheating: (**a**)–(**c**) consequent snapshots of the system, where the symbols are particles coloured in accordance to the $\lambda ^2$-value (see Supplemental Movie 1). (**d**) Evolution of the field $\lambda ^2(r,t)$ in the radial direction (1) shown in (**a**). (**e**) $\lambda ^2(\tau )$-profile in the propagating melting front during the nuclei growth. The red symbols are experimental points, the red solid line is the theoretical fit (8). The blue symbols are the fraction of 6-fold Voronoi cells in the plane of analysis, with the sudden drop indicating the structure is breaking.

**Figure 3:**
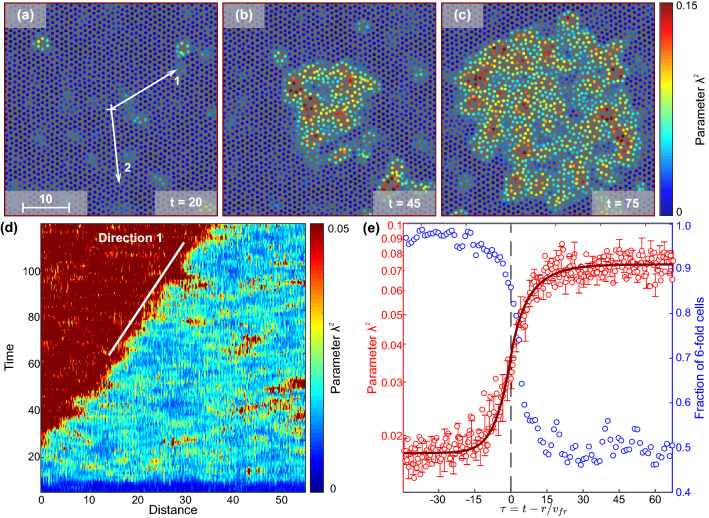
The self-similar $\lambda ^2$-profile of the propagating melting front in superheated bulk crystal observed with MD simulation: the description is the same as in Fig. 2. See also Supplemental Movie 2.

The results of evolution of $\lambda ^2$-field and of self-similar melting fronts in the NIPAm crystal are shown in Fig. 2. To study propagating melting fronts, the NIPAm colloidal fcc crystal was heated and a layer normal to [111]-direction was visualised. In this plane, the particles in fcc crystal are arranged in hexagonal ordered structure, that breaks on melting, as illustrated in Fig. 2a–c (see also Supplemental Movie 1). Here, the particles are coloured according to $\lambda ^2$-values calculated as explained in Eq. (2). Note that, contrary to Lindemann parameter, $\lambda ^2$ determined for a given structure has a finite value both in crystal and fluid, and is insensitive to the loss of particles moving in and out of the layer under analysis.

We analysed the evolution of $\lambda ^2$-parameter at different distances along the direction (1) in Fig. 2a, in the same manner as reported in Refs.^44,48^. Evolution of $\lambda ^2$ in the direction of interest 1 is shown in Fig. 2d. Here, one can see formation of the liquid nuclei with radius $\simeq 15\,{\upmu {\hbox {m}}}$ and its growth, as indicated by transition from the blue- to the red-coloured region in $\lambda ^2$. The large size of the nucleus in the experiment is explained by the almost simultaneous occurrence of several closely located small nuclei illustrated in Fig. 2a with their subsequent merging. After this, the evolution of the nucleus is determined by Eq. (5). The crystal is assumed to be overheated uniformly over the volume, and the size of the system is much larger than the scale of the nuclei. This means that at large radii of the nuclei, the melting front is planar, whereas its constant velocity is supported by permanent “release of disorder” during melting front propagation (due to the gap in the Helmholtz energy), similar to reported in Refs.^44,48,58^. The solid white line corresponds to the melting front velocity $v_{\rm{fr}}\simeq 0.05\,{\upmu }$m/s.

The melting regime we just observed was reported in Ref.^13^ as the *intermediate superheating*. In this regime, a liquid nucleus grows in a manner similar to that at weak superheating (melting front propagates consistently, with rare “jumps” caused by nucleation before the front), but the front velocity already nonlinearly depends on the value of the volume fraction “superheating” $\Delta \phi = \phi _m - \phi$, where $\phi _m =54.5\%$ is the melting volume fraction. To test this, we used the particles with diameter of $\sim 1.33$ larger than those in Ref.^13^ ($1.04\,{\upmu {\hbox {m}}}$ versus $0.78\,{\upmu {\hbox {m}}}$ at $25^{\circ }{\hbox {C}}$), since $v_{\rm{fr}}$ is proportional to the particle size. Taking into account correspondence between experiments with particles of different sizes, we obtain $\Delta \phi = \phi _m - \phi \simeq 3.5\%$ in our case (this corresponds to $v_{\text{fr}}\simeq 0.05/1.33 = 0.037\,{\upmu {\hbox {m/s}}}$ for smaller particles, see Fig. 3c in Ref.^13^). Besides, the $\lambda ^2$-field evolution in Fig. 2 clearly illustrates a set of features inherent to intermediate superheating, including spontaneous formation and disappearance of small (unviable) nuclei, as well as strong oscillations of the front in Fig. 2d induced by thermal fluctuations, whose contribution becomes significant for the system in vicinity of phase transition, in accordance with the results reported in Refs.^60,61^.

To obtain the self-similar $\lambda ^2$-profile from the data for subsequent comparison with our model, we averaged the time dependencies of $\lambda ^2(\tau ) \equiv \lambda ^2(t-r/v_{\rm{fr}})$ at different distances from the center marked with the cross in Fig. 2a (10 points, uniformly distributed along the line 1, from 20 to $30\,{\upmu {\hbox {m}}}$, results for the line 2 are shown in Fig. S2). The experimentally obtained profile for $\lambda ^2(\tau )$ is shown in Fig. 2e with red symbols. The blue symbols represent the number of 6-fold cells in the plane of analysis. The red solid line here is the self-similar profile obtained using Eq. (8), whose parameters $p_{1,2}$, and $\lambda _*^2$ were found with least squares fitting ($\lambda _1^2$ was obtained with analysis of the crystal before melting). The $\lambda ^2$-values corresponding to the crystalline, fluid, and threshold states are $\lambda _1^2 \simeq 0.015$, $\lambda _2^2 \simeq 0.07$, and $\lambda _*^2 \simeq 0.025$, respectively.

One can see that the theoretical self-similar profile (red line in Fig. 2e) agrees very well with the experimental data, strongly supporting the self-consistent $\lambda ^2$-model we proposed. The transition point (vertical dashed line in Fig. 2e) between the exponential branches of the $\lambda ^2$-profile shows excellent correlation with the onset of intensive drop in the fraction of 6-fold Voronoi cells in the plane of analysis, indicating the crystalline structure is breaking up and evolving from low- to high-$\lambda ^2$ states, as was explained in Fig. 1.

### Direct observation of self-similar profile of steady melting fronts in MD simulations

The propagation of melting fronts is a slow process compared to the characteristic time of individual particle motions. That means that the description in terms of slowly-fluctuating $\lambda ^2$-field should be suitable both in colloids, exhibiting Brownian regime of individual particle motions, and in systems with Langevin dynamics of particles. To test whether the same picture, as we observed in colloids, can be found in atomic crystals, we used MD simulations with Langevin thermostat and weak damping. Under the conditions of our MD simulations (see "Materials and methods"), the system density at the melting and crystallisation (in dimensionless units) points is $n_m =0.93$ and $n_f = 0.88$, respectively^62^. Therefore, the stepwise-like change in particle diameter in our simulations can be estimated as $(n_f/n)^{1/3}-1\simeq 0.5\%$ ($n = 0.867$), from where one can estimate the drop in effective volume fraction from its melting value as $\Delta \phi \simeq (n_m/n)^{1/3}-1\simeq 2.4\%$. This value is close to the intermediate regime of superheating discussed in Ref.^13^. Note that the relationship between the superheating regimes in hard-sphere-like colloids and systems of particles interacting with more soft potentials stands beyond the scope of the present paper and should be studied in future.

The results of our MD simulations of the self-similar melting fronts in superheated bulk crystal of IPL18 particles are presented in Fig. 3 (the results for line 2 are provided in Fig. S3). We see that the parameters of the $\lambda ^2$-profile in Fig. 3e have slightly changed, compared to Fig. 2e. Here, the obtained $\lambda ^2$-values in the crystalline, fluid, and threshold states are $\lambda _1^2 \simeq 0.01$, $\lambda _2^2 \simeq 0.07$, and $\lambda _*^2 \simeq 0.035$, respectively. We see that, despite the fundamentally different dynamic regimes, spatial and time scales, inherent to colloids and atomic systems, the results of our MD simulations demonstrate striking similarity to the colloidal experiment. Even fluctuations of the melting front in simulations are very similar to those in experiments and agree with previous studies^13^ in the framework of the $\lambda ^2$-approach we proposed.

Despite significant fluctuations of $\lambda ^2$-field near the melting front, the mean-field description still holds. This is clear from a comparison of the mean interparticle distance and the characteristic space-scale of $\lambda ^2$-field fluctuation. In our experiment, $r_0 \simeq d_H \simeq 1.04\,{\upmu {\hbox {m}}}$ and is determined by the colloidal particle diameter, whereas the correlation length during the propagation of the melting front is $r_c \simeq v_{\rm{fr}}/p_c$ (here, $p_c\simeq {\min }\{p_1,p_2\}$ is the minimal exponential rate in Eq. (8)). Note that in the limit of small $v_{\rm{fr}}$, taking Eq. (8), we have $r_c = v_{\rm{fr}}/p_c\simeq \sqrt{{\chi }/{\gamma }}$: this characteristic (“diffusive”) length also follows from Eqs. (5) and (6). In the experiment, we have $v_{\rm{fr}} \simeq 0.05\,{\upmu }$m/s, $1/p_c\simeq 80$ s, from where $r_c=v_{\rm{fr}}/p_c \simeq 4\,{\upmu {\hbox {m}}}$. Note that $r_c$ is related to the characteristic spatial scale of the melting front fluctuations. Thus, considering that $r_0\simeq 1\,{\upmu {\hbox {m}}}$, we have $r_c/r_0\simeq 4$ for our experiment. For MD simulations, we have (in dimensionless units) $v_{\rm{fr}} \simeq 0.37$, $1/p_c\simeq 10$, and $r_0 = a^{1/18} \simeq 1.05$ (here, $a=2.365$, see "Materials and methods"), from where $r_c=v_{\rm{fr}}/p_c \simeq 3.7$ and $r_c/r_0 \simeq 3.52$. Therefore, it is clear that the relation $r_c/r_0$ is moderately large, allowing us to use the mean-field description for both colloidal experiments and the MD simulations we performed.

### Nucleation behaviour and bifurcation of $\lambda ^2$-field

The nucleation process of melting is known to consist of three stages^13^: (i) incubation of superheated crystal in a metastable state before formation of critical nuclei (e.g., defects^63^ or particle self-diffusion loops^64^), (ii) formation of critical nuclei^32,65,66^ and (iii) the growth of post-critical nuclei (this can be seen in Figs. 2 and 3). During a phase transition, formation of a critical nuclei is known to be realised through intermediate states (or activated clusters)^57^. After the clusters are formed, the system evolves between two states with the structure fluctuations playing a crucial role. The proposed $\lambda ^2$-model (5) predicts propagation of self-similar fronts corresponding to the third stage of nucleation process with the $\lambda ^2$-profile (8) consisting of two exponential branches. Formation of propagating melting fronts in overheated crystals and the double-exponent $\lambda ^2$-profile are observed in colloidal experiments (with Brownian dynamics of particles) and in MD simulations (with Langevin dynamics of particles). Here, we provide detailed numerical analysis of the first two stages: evolution of different initial $\lambda ^2$-fluctuations (nuclei), as well as spontaneous formation of critical nuclei (homogeneous nucleation), as described by the $\lambda ^2$-model. Hereby, the developed model will be shown to describe self-consistently all stages of the nucleation process, including essentially nonlinear first two stages.

We have seen that $\lambda ^2$-fluctuations, provided in Eq. (5) by thermal noise source $\xi (t,\mathbf {r})$, affect the melting front propagation. This is caused by high susceptibility of the system to the fluctuations in the vicinity of phase transition. The transition occurs at $\lambda _{\rm{cr}}^2 = \lambda _*^2$, and some of the fluctuations in the vicinity of the melting fronts affect the front propagation, as highlighted in Figs. 2d and 3d. However, the $\lambda ^2$-fluctuations become more important at the initial stages of nucleation: Weakly or strongly spatially localised (subcritical) fluctuations vanish, whereas sufficiently strong $\lambda ^2$-fluctuations (activated clusters) can transform to the nucleus of fluid state. The generation of the activated nuclei, their development and collapse during their evolution demonstrates *bifurcation behaviour*, since even weak change in parameters of the nuclei near corresponding critical values results in drastic and qualitative difference in the dynamics of their evolution.

To illustrate and study nontrivial bifurcation behaviour of the model (5), sensitive to the effects of thermal noise and structure of initial $\lambda ^2$-distribution (nuclei), we considered the stochastic differential equation (SDE). Following from Eqs. (5) and (6) we write:

9 $$\begin{aligned} \begin{aligned} \partial _t \lambda ^2&= \nabla ^2\lambda ^2 + Q (\lambda ^2) + \varepsilon ^{1/2}\xi (t,\mathbf {r}), \\ Q(\lambda ^2)&= -(\lambda ^2-\lambda _1^2) + (\lambda _2^2-\lambda _1^2)\eta (\lambda ^2-1), \end{aligned} \end{aligned}$$

where we have normalised $\lambda ^2$ to $\lambda _*^2$, $\lambda ^2/\lambda ^2_*\rightarrow \lambda ^2$, time $t\gamma \rightarrow t$, and distances $r \sqrt{\gamma /\chi } \rightarrow r$, assuming for simplicity that $\chi _{1,2}=\chi$, $\gamma _{1,2} = \gamma$; $\eta (s)=(1+\exp (-100 s ))^{-1}$ is a smoothed Heaviside step function, and $\left<\xi (t,\mathbf {r})\xi (t',\mathbf {r}')\right>=\delta (t-t')\delta (\mathbf {r}-\mathbf {r'})$. One can see that the free parameters in Eq. (9) are the (normalised) thermal noise magnitude $\varepsilon$ and the normalised parameters $\lambda ^2_{1,2}$. For modeling, we used experimentally obtained $\lambda _1^2 = 0.6$ and $\lambda _2^2 = 3.0$.

The proposed model takes into account several physical effects described by different terms in Eq. (9). The first term is the diffusion of the $\lambda ^2$-field, facilitating its relaxation into a homogeneous $\lambda ^2$-distribution – crystalline or fluid, depending on the domains to which the system belongs. From the physical point of view, this gradient term is related to the creation of a new surface during nucleation. The diffusive term tends to homogenise the system, preventing the creation of new surfaces requiring excess positive energy. The second term in Eq. (9) is related with the barrier in the free energy during the phase transition, as illustrated in Fig. 1c: while a fluctuation is weak and insufficient to overcome the energy barrier, $Q(\lambda ^2)$ favours the same state, whereas a strong $\lambda ^2$-fluctuation can induce the transition from crystal to fluid. Thus, the $Q(\lambda ^2)$-term takes into account the activation nature of nucleation. The last, noise term in Eq. (9) describes generation and annihilation of fluctuations—thermal “breathing” of the system due to collective excitations. As it has been pointed out above, thermal fluctuations play an exceptionally important role in vicinity of phase transitions. At the same time, the resulting dynamics of melting is governed by different factors related with creation of new solid-fluid surface, free energy release, and thermal collective fluctuations. A complicated interplay between these factors leads to essentially nonlinear evolution of fluid nuclei in overheated crystals, characterised by bifurcation in their dynamics that depends on initial conditions.

To illustrate bifurcation behaviour resulting in nucleation and formation of steady melting fronts, we solved Eq. (9) and considered evolution of $\lambda ^2$-field with the Gaussian initial distribution:

10 $$\begin{aligned} \lambda ^2(0, \mathbf {r}) = \lambda ^2_1 + \delta \lambda ^2 \exp \left( -\frac{\mathbf {r}^2}{l^2}\right) , \end{aligned}$$

where the magnitude $\delta \lambda ^2$ was varying in the range from 0 to 1.5, and we considered distribution (10) with $l^2=0.4$ in 1D and $l^2=1$ in 2D case. At the boundaries of the systems $\lambda ^2 = \lambda _1^2$ was kept fixed, while the system size was chosen to be much larger compared to *l* (20 and $31.62 \times 31.62$ in 1D and 2D case, respectively). Equation (9) with initial distribution (10) was solved with exponential Euler scheme^67^, using the timestep of $\Delta t=10^{-3}$ ($\Delta t=5\times 10^{-4}$) and 2048 ($512\times 512$) eigenfunctions in 1D (2D) case. In 3D case, we considered the system with the sizes $31.62 \times 31.62 \times 31.62$, using the timestep $\Delta t=5\times 10^{-4}$ and $128\times 128\times 128$ eigenfunctions.

**Figure 4:**
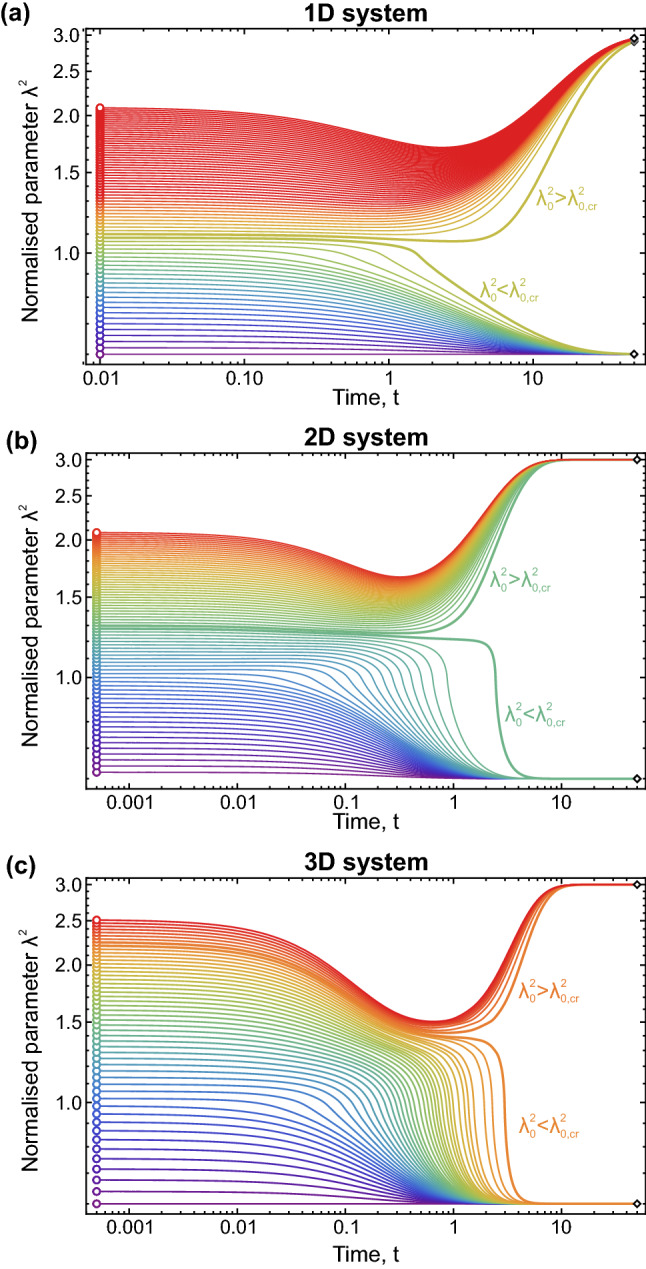
Bifurcation behaviour of different initial fluctuation (10) given with self-consistent model of $\lambda ^2$-evolution: Dependencies of $\lambda ^2(t,0)$ at different initial value $\lambda ^2(0,0) \equiv \lambda _0^2 \gtrless \lambda _{0,\hbox {cr}}^2$ are shown for (**a**) 1D, (**b**) 2D, and (**c**) 3D Gaussian nuclei. Bifurcation behaviour is clearly justified with qualitative different $\lambda ^2(t,0)$-behaviour at small change in the initial state in vicinity of $\lambda _{0,\hbox {cr}}^2$.

One should note that, speaking about the dimensions of the problem, we refer to the symmetry of the nuclei. As such, the equations are written in the same form for planar or bulk melting systems and the difference is related to the form of operators $\nabla ^2$ in each case in Eq. (9). Thus fluctuation (10) in 1D case is a plane, and we can speak about melting initiated by a heated grain. The 2D case means the nuclei is of cylindrical form, whereas 3D case corresponds to the spherical nuclei.

The results at $\varepsilon = 0$ in the case of 1D, 2D, and 3D Gaussian nuclei (10) are presented in Fig. 4. The time dependencies $\lambda ^2(t,0)$ are shown for different initial magnitudes of $\delta \lambda ^2$ of the $\lambda ^2$-distributions (clusters) that characterise their initial activation. One can see that, depending on the magnitude $\delta \lambda ^2$ (or $\lambda _0^2 \equiv \lambda ^2(0,0)=\lambda _1^2 + \delta \lambda ^2$), the solution exhibits bifurcation behaviour, with critical values $\lambda ^2_{0,\hbox {cr}} \simeq 1.09$ in 1D case, $\lambda ^2_{0,\hbox {cr}}\simeq 1.29$ in 2D, and $\lambda ^2_{\rm{cr}}\simeq 2.2$ for 3D nuclei. As clearly seen in Fig. 4, the fluctuations with $\lambda _0^2 < \lambda ^2_{0,{\rm{cr}}}$ vanish, the system tends to the low-$\lambda ^2$ (crystalline) state, whereas the ones with $\lambda _0^2 > \lambda ^2_{0,{\rm{cr}}}$ evolve to high-$\lambda ^2$ (fluid) state. Note, that for $\lambda _0^2 > \lambda ^2_{0,\hbox {cr}}$ the bifurcation behaviour also depends on the initial value of $\lambda _0^2$ and this is most obvious for the upper curves in Fig. 4a–c where one can clearly see initially a decrease in the value of $\lambda _0^2$ followed by a steep rise as the system enters the fluid state. This initial drop reflects energy transfer to the neighbours from the central particles in initial $\lambda ^2(0,0)$ site. We see that the largest initial $\lambda ^2$-fluctuation, capable of inducing phase transition, corresponds to the 3D nuclei. This is expected, since the nucleus formation is governed by interplay between surface formation processes (related to $(\nabla \lambda ^2)^2$ term in Eq. (4)) and free-energy release (described by the source $Q(\lambda ^2)$ during the evolution of the system).

**Figure 5:**
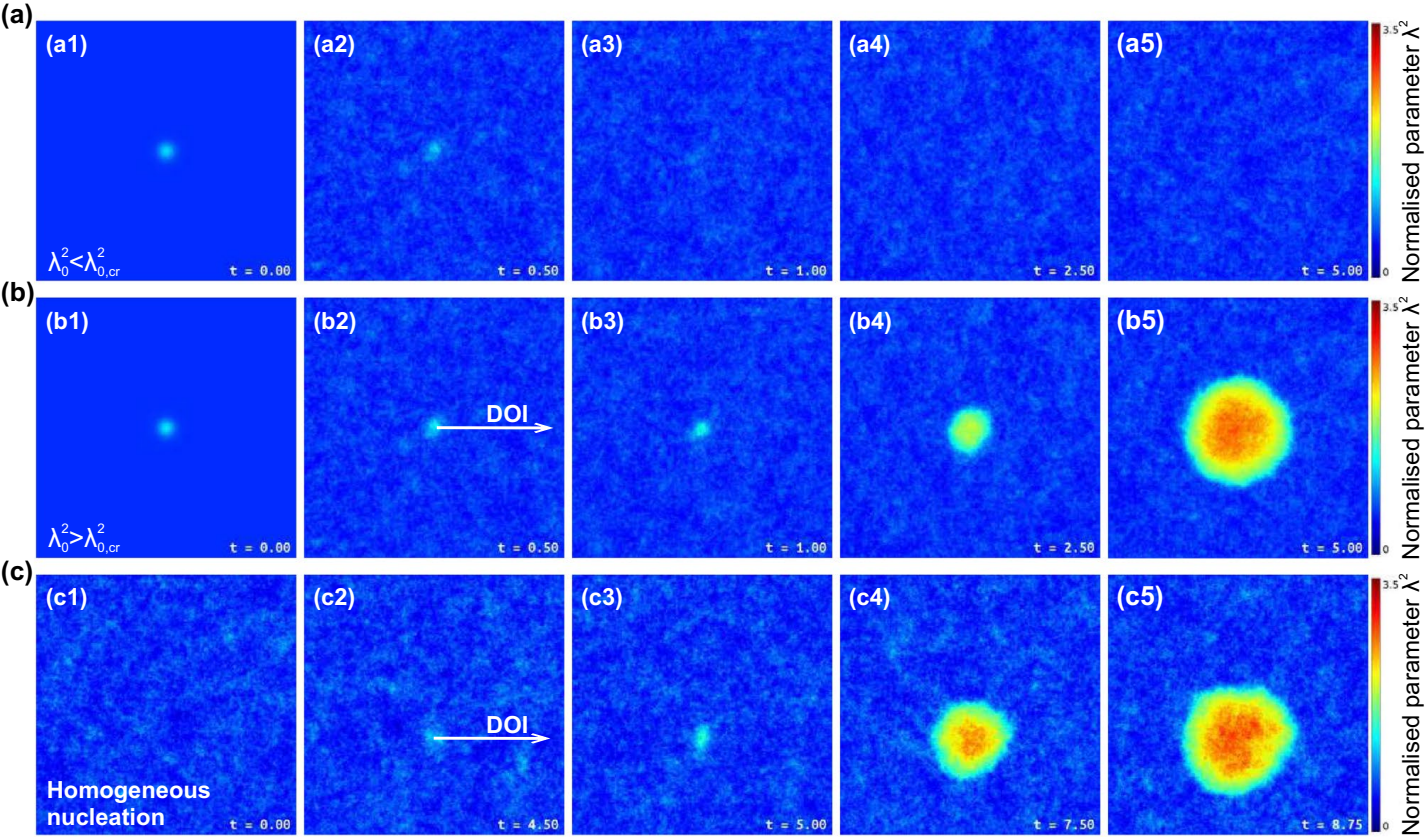
Evolution of different initial 2D (cylindrical) $\lambda ^2$-fluctuations (nuclei), and spontaneous nucleation in homogeneous system: Sequential frames illustrate the evolution of (**a**) subcritical and (**b**) supercritical $\lambda ^2$-fluctuation (nuclei), and (**c**) spontaneous nucleation due to thermal noise. For details see Supplemental Movies 3–5.

**Figure 6:**
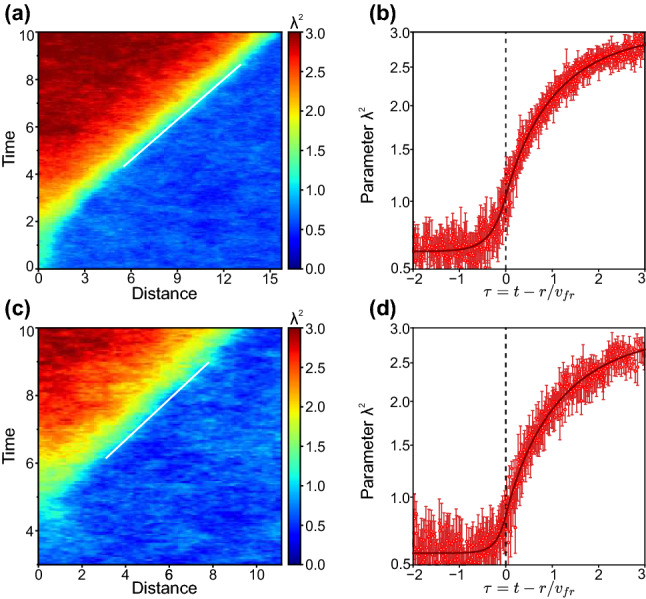
Self-similar fronts during the growth of supercritical and spontaneously-formed nucleus: Evolution of $\lambda ^2$-field in radial directions shown with white arrows in Fig. 5(b2, c2) for (**a**,**b**) supercritical and (**c**,**d**) spontaneously-formed nucleus. (**a**,**c**) and (**b**,**d**) are obtained in the same manner as (**d**,**e**) in Figs. 2 and 3.

Weak thermal noise affects slightly behaviour of the near-critical initial states (see Fig. S4 in Supplementary Materials). The critical values $\lambda ^2_{0,\hbox {cr}}$ depend on the particular choice of the fluctuation profile (related to $\lambda ^2$-gradients) and (slightly) on the dimension, because of the bifurcation problem is essentially nonlinear: The resulting scenario of $\lambda ^2$-evolution is governed by interplay of $\lambda ^2$-generation and dissipation in Eq. (9) and, in general case $\lambda _{\rm{cr}}^2 > \lambda _*^2$ due to the curvature of the spatially-inhomogeneous $\lambda ^2$-fluctuation (nuclei).

The effects of the thermal noise are illustrated in Fig. 5 with the results obtained for different initial fluctuations (nuclei), as well as for the case of spontaneous (thermally-induced) nucleation in homogeneous system. Here, Fig. 5a, b demonstrate snapshots of the nuclei with $\lambda ^2_0 \lessgtr \lambda ^2_{0,\hbox {cr}}$ (see also Supplemental Movies 3 and 4). These results correspond to the cases $\lambda _0^2 =1.28$ and 1.3 in Fig. 4b at $\varepsilon = 10^{-4}$. The initial conditions of the simulation are shown in Fig. 5(a1, b1), while the evolution of the systems is illustrated with Fig. 5(a2–a5, b2–b5) (see also Supplemental Movies 3 and 4). One can see that the thermal noise can affect the shape of the near-critical nucleus and the form of a melting front, as highlighted in Fig. 5(b4). However, as the nucleus evolves it becomes symmetric as seen in Fig. 5(b5) and in Supplemental Movie 4. The same behaviour was observed in experiments on liquid nucleus growth in homogeneous melting of colloidal crystals^13^.

The spontaneous nucleation process in initially-homogeneous system is illustrated in Fig. 5c and Supplemental Movie 5. Note that these results illustrate numerical solution of Eq. (9), and we considered a homogeneous state with $\lambda _0^2 = 0.6$ and $\varepsilon =5.8\times 10^{-4}$. We see that under sufficiently strong thermal noise, fluctuation mechanism provides *spontaneous formation* of critical nucleus, as illustrated in Fig. 5(c3). The growth of a nucleus is accompanied by formation of a liquid nuclei before the melting front, in the same manner as we have seen in experiment and MD simulations (see Supplemental Movies 1 and 2).

**Figure 7:**
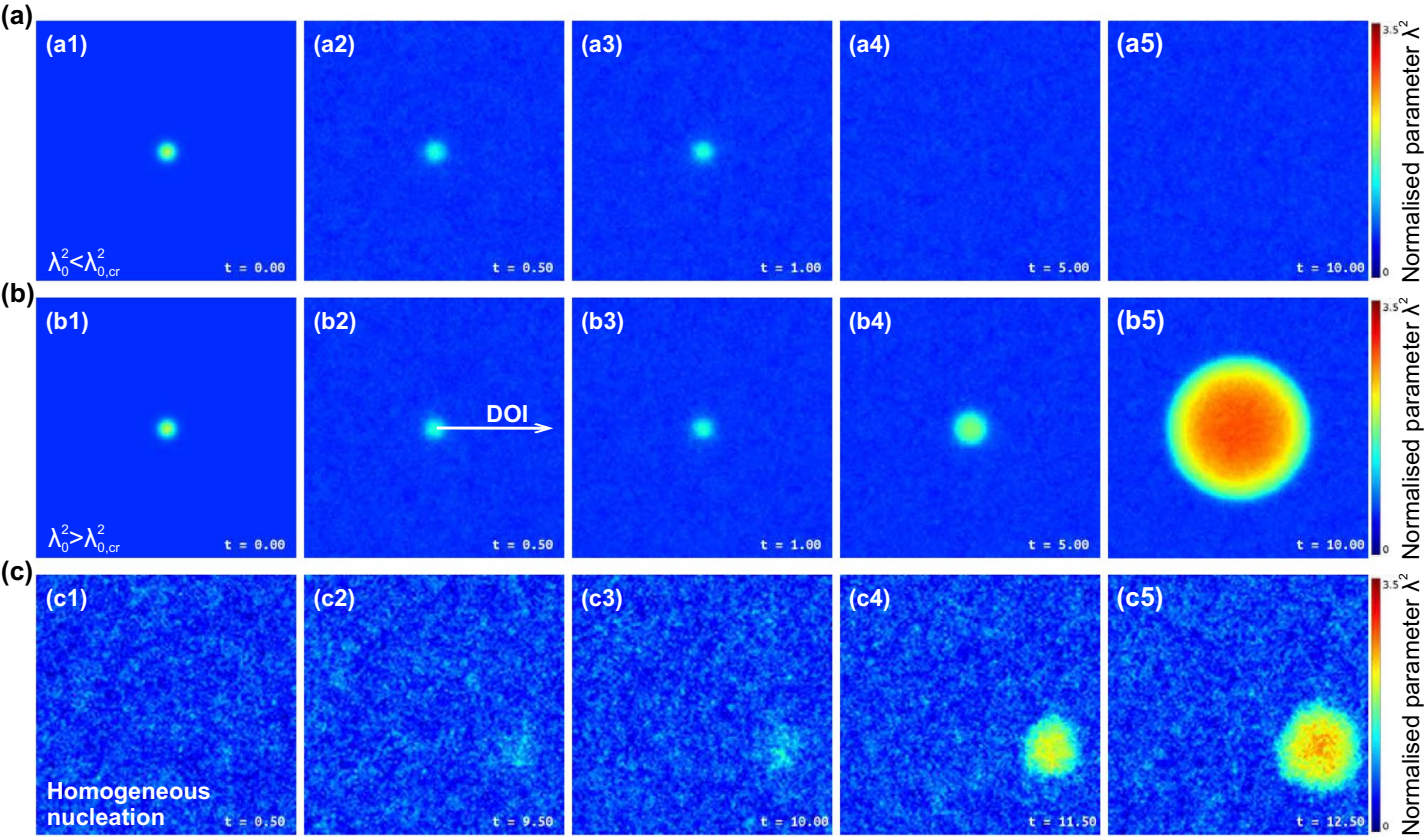
Evolution of different initial 3D (spherical) $\lambda ^2$-fluctuations (nuclei), and spontaneous nucleation in homogeneous system: sequential frames illustrate the evolution of (**a**) subcritical and (**b**) supercritical $\lambda ^2$-fluctuation (nuclei), and (**c**) spontaneous nucleation due to thermal noise (see also Supplemental Movie 6).

**Figure 8:**
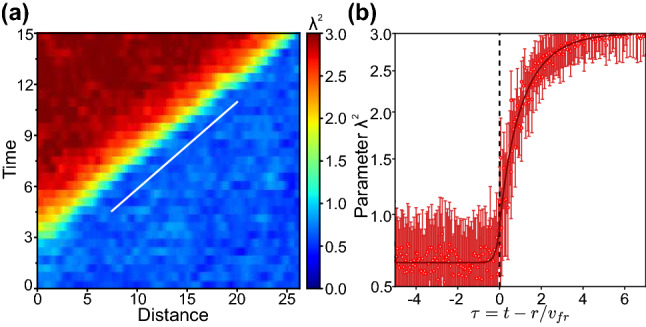
Self-similar front during the growth of nucleus in 3D system: evolution of $\lambda ^2$-field in radial directions shown with white arrows in Fig. 7(b2) for supercritical nucleus. (**a**) and (**b**) are obtained in the same manner as (**d**,**e**) in Figs. 2 and 3.

We processed the $\lambda ^2$-evolution of the data shown in Fig. 5b, c, in the directions of interest (indicated in Fig. 5 with white arrows), in the same manner as we have done with the experimental and MD data shown in Figs. 2 and 3. The results illustrated in Fig. 6a, b correspond to the growth of the critical nucleus shown in Fig. 5b. One can see in Fig. 6a formation of the melting front from the initial nucleus. Note that the same behaviour was observed experimentally in complex plasmas, as reported in Ref.^45^, supporting the analogy we found here.

After the melting front is formed, the $\lambda ^2$-profile is described by two exponential branches (solid red line in Fig. 6b) as we now expect. In the same manner, the results for homogeneous nucleation (Fig. 5c and Supplemental Movie 5) are shown Fig. 6c,d. In this case, the $\lambda ^2$-field strongly fluctuates due to the enhanced thermal noise and the melting front is slightly more smeared spatially (as highlighted with green color band in Fig. 6c), but the general behaviour is the same as in Fig. 6a, b. This is where self-consistency of the proposed model becomes particularly evident: experimental results shown in Figs. 2 and 3 and described by Eq. (5) are recovered in Figs. 5 and 6 using Eq. (9).

The trends we just observed for evolution of 2D nuclei are qualitatively the same in 3D case where we consider spherical nuclei, as illustrated in Figs. 7 and 8. Here, the cross-sections of the system are shown in Fig. 7a, b, to illustrate the nucleation of sub- and supercritical nuclei at $\lambda _0^2=2.21$ and $\lambda _0^2=2.23$, respectively. The general picture of the cluster evolution is completely the same as in 2D case. The case of homogeneous nucleation is shown in Fig. 7c (see also Supplemental Movie 6), where one can see that strong thermal fluctuations can form an activated cluster, capable of inducing a phase transition through propagating melting fronts. The latter is illustrated in Fig. 8 in the same manner as in Fig. 6a, b. One can see in Fig. 8a that after the nucleus is formed, it grows linearly. The $\lambda ^2$-profile at large distances from the center of nucleation was calculated in the same manner as those in Figs. 2, 3, and 6, and the result is shown in Fig. 8b: again, the $\lambda ^2$-profile consists of two-exponent branches (shown by solid red line), in complete agreement with our experimental and MD results discussed previously. Thus, the proposed mean-field model consistently describes nucleation and bifurcation behaviour of $\lambda ^2$-field in 1D, 2D and 3D systems.

## Conclusions

In this work, we proposed a mean-field model of melting based on the new order parameter we developed: $\lambda ^2$—mean-square displacement reformulated to include particles in neighbouring Voronoi cells. The key element of reformulation is use of Voronoi cell construction around a particle and folding in the contributions from the neighbouring cells. Behaviour of $\lambda ^2$-field was analyzed using a time-dependent Langevin equation with thermal noise and source terms. We show that the model we developed exhibits essentially nonlinear behaviour, while the terms have a clear physical meaning when applied to the analysis of crystal melting. This has been demonstrated by analysis of the experimental data using systems with significantly different dynamic regimes—Brownian in colloids and Newtonian in MD—with the model demonstrating excellent description of propagation of melting fronts and their structure. Furthermore, being intrinsically microscopic, the proposed model allows to study in details nucleation in different regimes (depending on the magnitude of thermal noise) of superheating, as well as evolution of realistic liquid nuclei that can assume a variety of complicated shapes.

With experimental study of melting of NIPAm colloidal crystals and MD simulations of superheated hard-sphere-like crystals of IPL18 particles, we proved that the $\lambda ^2$-profile of the steady fronts consist of two exponential branches. Moreover, we demonstrated that the proposed model exhibits bifurcations and behaviour inherent to the initial stages of nucleation process and allows to recover completely the process of nucleation in a homogeneous system and the melting front kinetics. To prove this, in addition to comparison with experiment and MD simulations, we studied the evolution of sub- and supercritical planar (1D), cylindrical (2D), and spherical nuclei (3D), as well as homogeneous nucleation in 2D and 3D systems. Remarkably, we found that our model provides a clear analogy between the melting fronts in superheated colloidal and atomic crystals and non-equilibrium melting in complex (dusty) plasmas as well as with the reaction (activation) fronts in exothermic chemically-reactive media, suggesting that the proposed theoretical framework is suitable for a wide range of phenomena, from atomic and molecular to colloidal and globular protein systems.

Nucleation process in superheated crystals, kinetics of formation and growth of liquid nuclei, and structure of steady melting fronts represent central problems for understanding crystal melting. The proposed model provides the first approximation for description melting in overheated crystals. As the next steps, anisotropic character of parameters used in the model should be taken into account. Fundamentally, this is related to anisotropy of properties (e.g., surface energy, elastic modulus), susceptibilities, and relaxation kinetics in crystals. However, already in our simple isotropic approximation, we see that the $\lambda ^2$-based model describes nucleation, formation, and propagation of melting fronts, thus, opening a way for detailed future studies of these phenomena. We believe that the presented results make an essential advance providing a simple and effective tool for study of nucleation process and melting in superheated crystals of different nature, that should be of interest to the broad community in condensed matter, materials science, chemical physics, and soft matter.

From the experimental point, $\lambda ^2$ is directly related to the second cumulant of the first peak in a pair correlation function, representing an important advantage for future experiments with typical atomic systems where $\lambda ^2$ can be obtained from, for example, X-ray or neutron scattering data. The corresponding field of second cumulants could also be extracted experimentally using, for example, extended X-ray absorption fine structure (EXAFS)^68–73^. This presents an exciting opportunity to measure $\lambda ^2$ evolution experimentally in real materials providing a route to study microscopic picture of melting, including under challenging environmental (e.g. extreme) conditions.

Here, we considered self-similar profiles of propagating melting fronts. However, the $\lambda ^2$-model we developed can be applied to analyse the fractal dimensions of the clusters of “hot” particles (with relatively large $\lambda ^2$) during nucleation in different regimes of overheating. In this case, the power exponent could be identified with analysis of the cluster sizes distributions, similar to those reported in Ref.^17^. Besides, there is a number of other interesting problems, related to melting that stand beyond the scope of our present paper, but deserving separate studies. The first one is the problem of local inversion-symmetry breaking during melting. Since the $\lambda ^2$-parameter operates with MSDs between the nearest neighbours, the $\lambda ^2$-approach does not distinguish centerosymmentric and noncentrocymmetric clusters if they are regular. However, $\lambda ^2(T)$ behaviour is determined by the local structure and can have different values at melting point, depending on the crystal symmetry and structure. The relation between $\lambda ^2(T_m)$, particular crystalline structure, and melting conditions should be studied in future. Another problem is related to the Lindemann and Born criteria of melting, formulated for MSDs of particles and shear modulus (at zero-frequency), respectively. We note that contrary to the Lindemann parameter formulated for MSDs of individual particles, $\lambda ^2$ is related to the *relative* MSDs between *the nearest neighbours*, i.e. it is closely related to the modified Lindemann criterion of melting introduced for 2D systems in Ref.^5^. As an order parameter, $\lambda ^2$ is not completely free parameter: in equilibrium, $\lambda ^2$ is determined by thermodynamic parameters (pressure, temperature, and density) of the system that minimise the free energy. Similarly, the zero-frequency shear modulus *G* is also determined unambiguously under given thermodynamic conditions. However, the temperature dependencies $\lambda ^2(T)$ and *G*(*T*) at a given density of a crystal are determined by the crystal structure. Therefore, the parametric dependencies in the plane $\{\lambda ^2, G\}$ could shed light onto the possibility of unification between Lindemann melting, Born melting, and the symmetry analysis.

Even more broadly, the proposed model opens rich prospectives for studies of melting as well as solidification, statistical analysis of nucleation process, nucleation at different regimes of superheating, from weak to strong ones and, in particular, of fluctuation mechanisms responsible for acceleration of melting fronts in strongly superheated regime^13^. The proposed approach can be generalised to various cases, including the systems of anisotropic and active particles. Furthermore, due to its formulation, the proposed model is equally suitable for analysis of melting in glassy systems as well as of the glass formation mechanism — one of the long standing issues in the condensed and soft matter science. Corresponding theoretical and experimental investigations are important for understanding the role of diffusion and of thermal noise in transition from slow to fast propagating melting fronts and for nucleation process. We leave these interesting problems for future works.

In conclusion, we note about possible application of the $\lambda ^2$-based framework for shear-induced melting and crystallisation. This could be done in a manner, similar to Ref.^74^: one should use free-energy functional taking into account the shear-induced term, instead of Eq. (4). However, the problem of crystallisation is essentially more complicated compared to melting, due to multiple possible pathways of crystallisation and capability of multidomain structure formation. After a polycrystalline structure is formed, the evolution is governed by slow processes, interaction of defects, dislocations, and grain boundaries. To account for these phenomena, the proposed $\lambda ^2$-based approach should be developed further.

## Materials and methods

### Details of NIPAm experiment

To study melting in superheated crystals, we performed the experiments in the same manner as those in Ref.^32^. To create 3D stable colloidal crystals, we used thermal-sensitive NIPAm colloidal spheres suspended in an aqueous buffer solution with 1mM acetic acid. A small amount of non-fluorescent red dye, 0.2% by volume, was added to the sample for absorbing heat. The effective particle diameters linearly changes from $1.04\,{\upmu {\hbox {m}}}$ at $25^{\circ }\hbox {C}$ to $0.89\,{\upmu {\hbox {m}}}$ at $30^{\circ }\hbox {C}$ in water. The temperature dependence of the hard-sphere diameter $d_H(T)$ obtained with dynamic light scattering is provided in Fig. S1 in Supplementary Materials.

The colloidal sample was placed in a glass capillary channel of sizes $\sim 18 \times 3 \times 0.1 \,{\hbox {mm}}^{3}$ and annealed to form a polycrystal with only a few domains. The refractive index of the particles and solvent were matching so that we could have visualised a layer in the middle of the system using bright-field microscopy. With increase in temperature, induced by laser heating of the system, the volume fraction was dropped to melt the sample^11^. More details about the experiments are provided in Ref.^32^.

### Details of MD simulations

To compliment the experiments with colloids, wherein the particles move in Brownian (overdamped) regime, we performed MD simulations of crystals for systems with Langevin dynamics. NIPAm particles interact by hard-sphere like potential^13,32^. Therefore, we considered the system of particles interacting via the inverse-power-law (IPL18) potential as a simple model:

11 $$\begin{aligned} \varphi (r) = \epsilon a \left( \frac{\sigma }{r}\right) ^{18}, \end{aligned}$$

where $\epsilon$ and $\sigma$ are the strength and the characteristic range of the repulsion, respectively, and parameter $a = 2.365$ was introduced for convenience to simulate the stepwise change in the particle diameter. Note, usage of the Yukawa or penetrable sphere interactions model is also possible, but it should result in the same results since near the hard-sphere limit is modelled. We used the normalised temperature $T/\epsilon \rightarrow T$, distance $r/\sigma \rightarrow r$, particle density $\rho \sigma ^3/m\rightarrow n$, and time $t\sqrt{\epsilon /m\sigma ^2}\rightarrow t$ (here, *m* is the mass of the particle).

To analyse melting of the superheated crystal, we performed MD simulations of a system containing $N=7.2\times 10^4$ particles in *NVT* ensemble at $n = 0.867$ and $T = 1$. In the initial state, the particles were arranged in fcc lattice with horizontal (111)-plane. The sizes of the simulation regions in *x*, *y*, and *z*-directions were chosen so, that $L_x/L_z\approx 20.4$ and $L_y/L_z\approx 21.3$. The time step of $\Delta t=7.4\times 10^{-4}\sqrt{m\sigma ^2/\epsilon }$ was used for simulations with LAMMPS. To equilibrate the system, we simulated the system for $10^5$ time steps with $a = 7.224$; then, *a* was stepwise reduced to $a = 2.365$ and the following $4\times 10^5$ steps were used for analysis of melting in the crystal.

## Supplementary information

Supplementary Information 1.

Supplementary Information 2.

Supplementary Information 3.

Supplementary Information 4.

Supplementary Information 5.

Supplementary Information 6.

Supplementary Information 7.
