# Supplementary material for: Mean-field model of melting in superheated crystals based on a single experimentally measurable order parameter
Source: Sci Rep. 2021 Sep 9;11:17963. doi: 10.1038/s41598-021-97124-7 (PMC8429456; doi:10.1038/s41598-021-97124-7)
Supplement: Supplementary file 1 — Supplementary Information 1. [file 41598_2021_97124_MOESM1_ESM.pdf]

Mean-field model of melting in superheated crystals based on a single experimentally measurable order parameter (Supplementary Materials)

Nikita P. Kryuchkov,[1] Nikita A. Dmitryuk,[1] Wei Li,[2] Pavel V. Ovcharov,[1] Yilong Han,[2] Andrei V. Sapelkin,[3] and Stanislav O. Yurchenko[1]

[1] *Bauman Moscow State Technical University, 2nd Baumanskaya street 5, 105005 Moscow, Russia*
[2] *Department of Physics, Hong Kong University of Science and Technology, Clear Water Bay, Hong Kong SAR, China*
[3] *School of Physics and Astronomy, Queen Mary University of London, London E1 4NS, England*

(Dated: July 21, 2021)

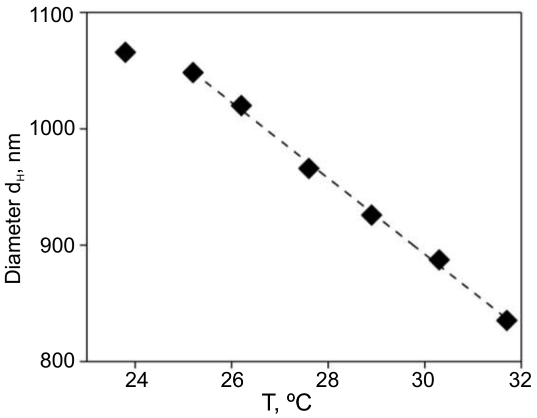

Figure S1. **Temperature dependence of the diameter $d_H(T)$ of NIPAm particles used in the present work:** The laser heating elicits the decrease in the particle diameter and volume fraction.

Supplemental Movie 1 – experimental observation of nucleation and formation of melting front in colloidal crystal.

Supplemental Movie 2 – MD simulation of nucleation and formation of melting front in superheated crystal.

Supplemental Movie 3 – evolution of 2D (cylindrical) subcritical λ^2-fluctuation (nuclei).

Supplemental Movie 4 – evolution of 2D (cylindrical) supercritical λ^2-fluctuation (nuclei).

Supplemental Movie 5 – spontaneous nucleation due to thermal noise (formation of 2D nuclei).

Supplemental Movie 6 – spontaneous nucleation due to thermal noise (formation of 3D nuclei).

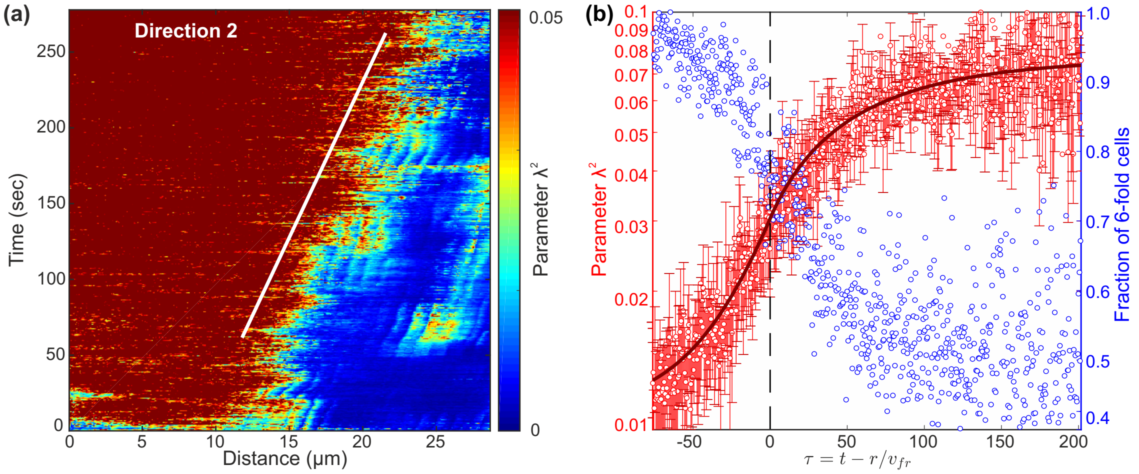

Figure S2. **Experimental self-similar profile of melting front:** (a) Evolution of λ^2 in the radial direction of interest (2) shown in Fig. 2(a) in the main text. (b) Self-similar profile $\lambda^2(\tau)$, the red symbols are experimental points, the red solid line is a fit given by Eq. (9) in the main text. The blue symbols are the fraction of 6-fold cells in the plane of analysis.

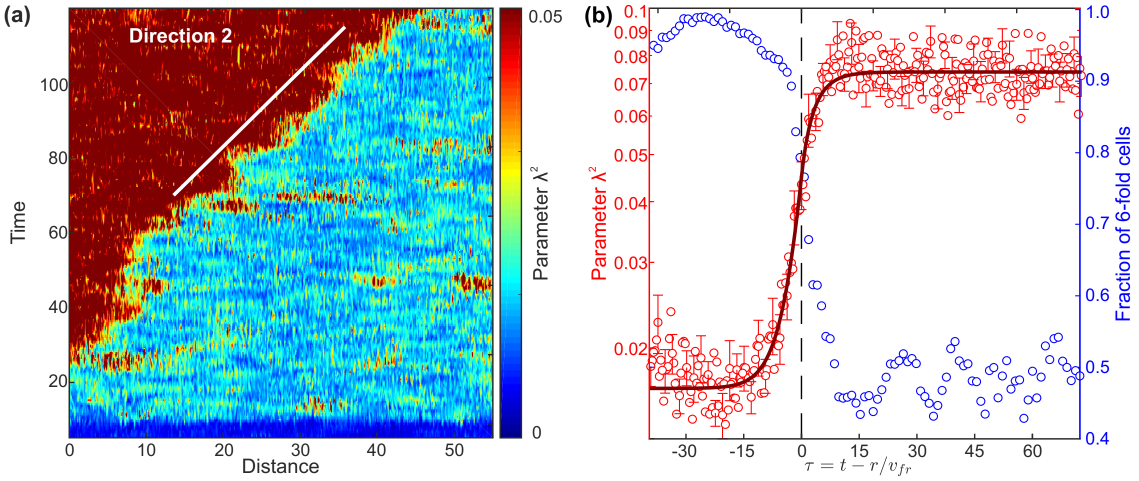

Figure S3. **MD self-similar profile of melting front:** (a) Evolution of λ^2 in the radial direction of interest (2) shown in Fig. 3(a) in the main text. (b) Self-similar profile $\lambda^2(\tau)$, the red symbols are MD points, the red solid line is a fit given by Eq. (9) in the main text. The blue symbols are the fraction of 6-fold cells in the plane of analysis.

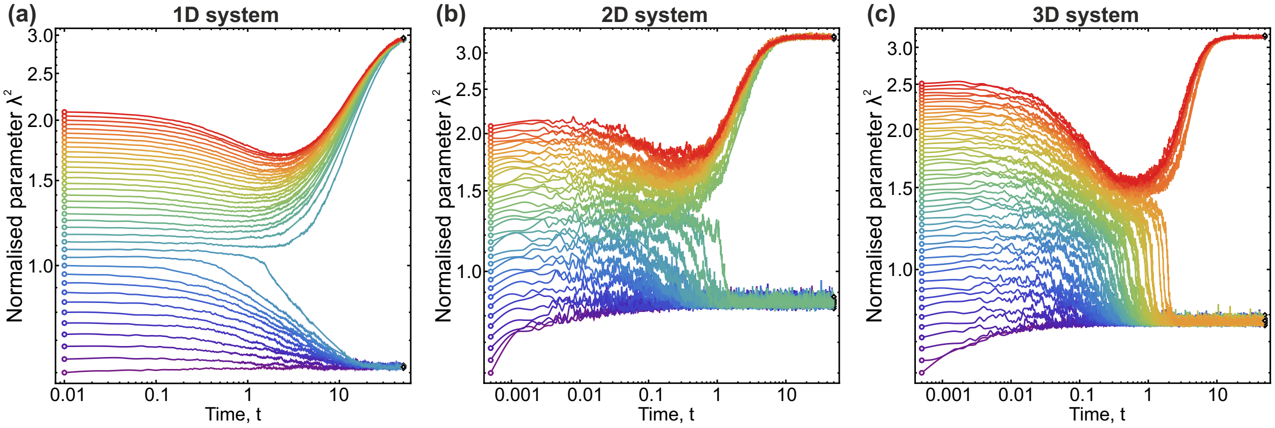

Figure S4. **The typical effect of thermal noise on the evolution of λ^2-field**: $\epsilon = 10^{-4}$ in 1D, 2D, and 3D cases shown in panels (a)-(c), respectively. Other description is the same as in Fig. 4 in the main text.
